# Decoding the adaptive survival mechanisms of breast cancer dormancy

**DOI:** 10.1038/s41388-025-03529-3

**Published:** 2025-08-27

**Authors:** Francis M. Barnieh, Jamie Morton, Olaitan Olanrewaju, Sherif F. El-Khamisy

**Affiliations:** 1 Institute of Cancer Therapeutics, Faculty of Life Sciences, University of Bradford, Bradford, UK; 2 School of Biosciences, Healthy Lifespan Institute, University of Sheffield, Sheffield, UK

**Keywords:** Breast cancer, Target identification

## Abstract

Breast cancer (BC) recurrence remains a major clinical challenge, leaving patients in perpetual uncertainty about disease relapse after primary treatment. BC dormancy, an adaptive survival state of disseminated tumour cells, is a key driver of both early and late recurrence. However, the mechanisms regulating BC dormancy remain poorly understood. Emerging evidence suggests that tumour hypoxia, extracellular matrix (ECM) remodelling, and therapy-induced stress drive dormancy by altering cellular metabolism, gene expression, and immune interactions, enabling long-term survival of dormant BC cells. With no dormancy-specific therapies currently approved, a deeper understanding of dormancy-associated survival mechanisms is crucial for identifying therapeutic targets and developing strategies to eradicate dormant BC cells, thereby preventing recurrence and improving patient outcomes. This review comprehensively examines major dormancy-inducing factors and the adaptive survival mechanisms of dormant BC cells. We also highlight critical gaps in preclinical models that hinder the translation of preclinical cancer dormancy insights into clinical applications and propose potential therapeutic strategies to prevent BC recurrence.

## Introduction

Despite significant advances in early diagnosis and treatment, breast cancer (BC) remains one of the most devastating malignancies worldwide, accounting for over 1 million deaths globally each year [1]. In the UK alone, BC accounts for about 11,500 deaths with ~56,000 new cases every year [2]. With current treatment advances, BC patients often respond to initial primary treatments (surgery ± radio and chemotherapy) with 76% attaining a 10-year survival rate in the UK [2]. However, despite the initial response to treatment, 25–45% of patients relapse with fatal secondary or metastatic disease at distant sites (e.g. lungs, liver, bone, brain, etc) months or years later [3, 4]. BC relapse/recurrence and distant metastasis account for >90% of all BC-related deaths and remain a significant challenge for curative treatment [4]. Once metastasis occurs in BC patients, the 5-year survival rate in the UK decreases from 86 to <20%, depending on the location of the metastases [2, 5].

BC is a heterogeneous disease with subtypes defined by the expression of hormone receptors: oestrogen (ER), progesterone (PR), and HER2 [6]. Although the effectiveness of BC primary treatment depends on the molecular subtype [7], the occurrence of BC relapse is independent of the disease subtype [4]. All BC subtypes can relapse post-treatment with aggressive metastatic recurrence. However, the site of recurrence, rate, and timing are subtype-specific. For example, the triple negative BC subtype characteristically relapses early ( < 5 years) [8], whilst relapse in the hormone receptor-positive subtypes occurs late (5–20 years) [3, 9]. Metastatic relapses remain a hallmark of incurable BC and occur when cancer cells from the primary tumour site migrate into secondary sites in multiple steps, often in a series of sequential cascading events before re-emerging as clinically detectable aggressive metastases [10]. Unfortunately, despite the clinical relevance of dormancy and its role in breast cancer relapse and recurrence, the biological mechanisms underlying these events remain largely elusive. Cancer dormancy has long been proposed to explain the late metastasis in patients, yet little is known about the survival mechanisms that sustain long-term dormancy [11, 12]. Cancer dormancy is the phenomenon where disseminated tumour cells (DTCs) from the primary tumour lie dormant and undetectable for many years, surviving in blood/lymphatic circulation before homing and re-proliferating at distant recurrence sites (the brain, bone, liver, lymph nodes, and lungs) [10]. This is often referred to as minimal residual disease (MRD) i.e. a population of dormant BC cells which are non-proliferative and resistant to anti-proliferative therapeutics [13, 14]. With no treatments available currently to target and eradicate dormant BC cells, the uncertainty about possible relapse after primary treatment remains a significant concern for patients in remission. Thus, understanding the mechanisms underlying cancer dormancy and subsequent metastatic recurrence remains crucial for identifying biomarkers and/or druggable targets to develop novel dormancy-specific therapeutics.

**Fig. 1:**
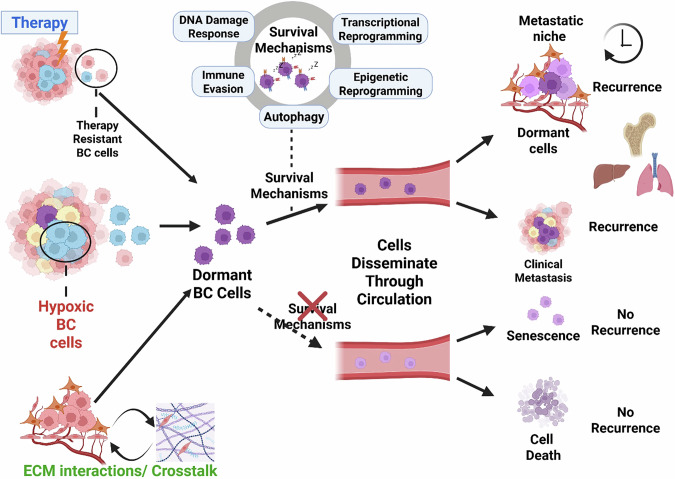
Overview of how breast cancer dormancy fuels recurrence. The emergence of dormant breast cancer (BC) cell populations occurs as an adaptive response to dormancy-inducing stressors within the primary tumour microenvironment, including hypoxia, extracellular matrix (ECM) remodelling, and anti-cancer therapies. Dormant disseminated tumour cells (DTCs) employ various survival mechanisms, mainly DNA damage repair, autophagy, immune evasion, and transcriptional and epigenetic reprogramming. These adaptations enable dormant DTCs to persist undetected in circulation while remaining biologically active. Upon reaching various metastatic niches, such as the bone (most common), lungs, liver, and brain, disseminated tumour cells (DTCs) may encounter specialised microenvironments that provide dormancy-supporting signals. These cues enable DTCs to persist in a quiescent state for extended periods. However, in response to reactivation signals such as inflammation, niche remodelling, or immunosuppression, DTCs can exit dormancy, resume proliferation, and give rise to overt metastases, ultimately leading to disease relapse. Although several potential therapeutic strategies have been suggested and are currently being attempted [185], we suggest that targeting the survival mechanisms sustaining BC dormancy holds significant promise for preventing recurrence.

This review, hence, aims to provide emerging insights into different processes and factors that promote dormancy and metastatic recurrence in BC and comprehensively discuss various adaptive mechanisms that sustain the long-term survival of dormant BC cells (Fig. 1). We conclude by providing insights into new strategies that may be exploited as therapeutic approaches for eradicating disseminated dormant breast cancer cells before the clinical recurrence of the disease.

## Breast cancer dormancy

Cancer dormancy was first proposed by Rupert Willis in 1934 to explain the late metastasis in patients where there was little evidence of local recurrence, suggesting that cancer cells may lay dormant in the tissues before recurrence [12]. These early observations were further expanded by Geoffrey Hadfield who suggested that recurrence occurred due to the dormant cancer cells entering “temporary mitotic arrest” [15]. The term “dormant” cancer cells describes the fact that these cancer cells remain alive in the tissues for long periods, halting proliferation while retaining the ability to reactivate and proliferate aggressively should conditions change. Dormant cancer cells are non-proliferating quiescent cells (albeit transcriptionally active) that have undergone G0-G1 cell cycle arrest with downregulated proliferation-related markers such as Ki67 and pRb [16] and high expression levels of dormancy-associated markers, including NR2F1 [17, 18].

It should be noted that tumour cell dormancy (cellular dormancy) as described above is different from tumour mass dormancy. Tumour mass dormancy is the steady state of tumour mass due to the balance between cell proliferation and death, with no apparent tumour progression [14, 19]. However, cellular dormancy (the focus of this review) is a slow-cycling or non-proliferative (quiescence) yet metabolically active state of cancer cells, which is more consistent with the classical definition of dormancy than tumour mass dormancy [20].

### Dormant cancer cells and cancer stem cells

Dormant cancer cells are often confused with cancer stem cells (CSC) such that the term dormant DTC and CSC are often used interchangeably. It is worth highlighting that although dormant DTC and CSC share common features, including the ability to remain quiescent, stem-like characteristics and therapy resistance, all of which are related, they are altogether a distinct population of cells. CSC are defined as cancer cells which can self-renew and differentiate, aiding the heterogeneity often found within tumours [21]. CSC also have an enhanced resistance to therapeutics whilst displaying the ability to evade the immune system, invade surrounding tissues, and metastasise to secondary sites [21]. Whilst these characteristics are very similar to those used to define dormant DTC, the key differences are that, firstly, CSC haven’t undergone cell cycle arrest and continue to cycle and divide albeit very slowly in contrast to the G0-G1 cell cycle arrest seen in dormant DTC [22, 23]. Secondly, CSCs express (by their very nature) stem-ness markers such as CD34, NANOG, etc, which dormant cancer cells do not express or express as a minimal selection of markers [14]. Thirdly, CSCs may retain the ability to differentiate into different cell types, unlike the dormant cancer cells, which remain at the same stage of differentiation and additionally represent a subpopulation that can perpetuate the growth of the malignant cell population indefinitely [14, 24–26].

### Cancer dormancy drive BC recurrence

Although the biological understanding remains elusive, cancer dormancy has now been well-established as a key culprit of BC relapse. Patients diagnosed with BC, regardless of the clinical stage, already harbour dormant disseminated tumour cells (DTCs) in distant metastatic sites, bone marrow, in particular [27, 28]. For example, almost 50% of BC patients with persistent DTCs in their bone marrow experience recurrence >5 years post-treatment [29]. Additionally, the increased number of DTCs found in bone marrow aspirates was found to correlate directly with reduced metastasis-free survival in BC patients [30]. Clinical evidence in both primary tumours and metastases suggests that late BC recurrence occurs as a result of dormant BC cells having the ability to survive silently in a state of dormancy and escape treatment, considering that conventional cancer therapies target proliferating cells [31].

Dormant DTCs in blood or lymphatic circulation are referred to as circulating tumour cells (CTCs). The detection of CTCs in BC patients has been shown to correlate with the incidence of metastasis and disease progression [28, 32, 33]. For example, a recent study in 177 women with metastatic BC demonstrated that the burden of CTCs in patients’ blood was directly related to disease progression and a poor prognosis [34]. In addition, CTCs have been detected in patients’ blood decades after primary treatment [3, 35]. These observations suggest that tumour cells exist in circulation in a state of dynamic dormancy, which fuels BC relapse [36].

Surprisingly, the detection of circulating BC cells has also been reported in healthy women without BC [33, 37]. For example, in a recent study, CTCs of BC were detected in 17.2% of healthy women with no BC diagnosis [33]. These observations support the narrative that cancer cells disseminate early in the form of dormant disseminated tumour cells (DTC) before an initial diagnosis of the disease. With the current limitations in detecting DTCs and CTCs, the clinical burden of DTCs and CTCs is thought to be underestimated in patients with many solid tumours, including BC [37]. Although the biological understanding of recurrence and metastatic BC remains limited, the recent evidence, as discussed, strongly implicates dormancy as a key culprit of the disease. Thus, a better understanding of the biological mechanisms underlying the establishment and maintenance of BC dormancy and recurrence is key to improving patients’ outcomes.

## Induction and establishment of dormancy in BC

The signals and factors that drive dormancy in cancers remain poorly understood, however, cellular dormancy of most cancers, including BC, is believed to be an induced adaptive and protective state of cancer cells in response to primary tumour microenvironment stresses (hypoxia, reduced access to nutrients), extracellular matrix (ECM) interactions, and anti-cancer therapeutics [17, 38–40]. Thus, cancer dormancy is considered a protective state adopted by cancer cells to promote survival whilst evading treatment. Recent and expanding evidence strongly suggests that the microenvironment surrounding both DTCs and CTCs is critical in establishing and maintaining this cell population [41]. Considering the scarcity of experimental models which accurately mimic clinical dormant phenotypes, characterised by low or absent expression of proliferation markers (e.g., Ki-67, PCNA), upregulation of dormancy-associated genes (e.g., NR2F1, DEC2, p27), resistance to chemotherapy, and altered metabolic and signalling pathways, the understanding of how dormant BC cells are induced and sustained remains critical for developing clinically-relevant experimental models.

### Tumour hypoxia

**Fig. 2:**
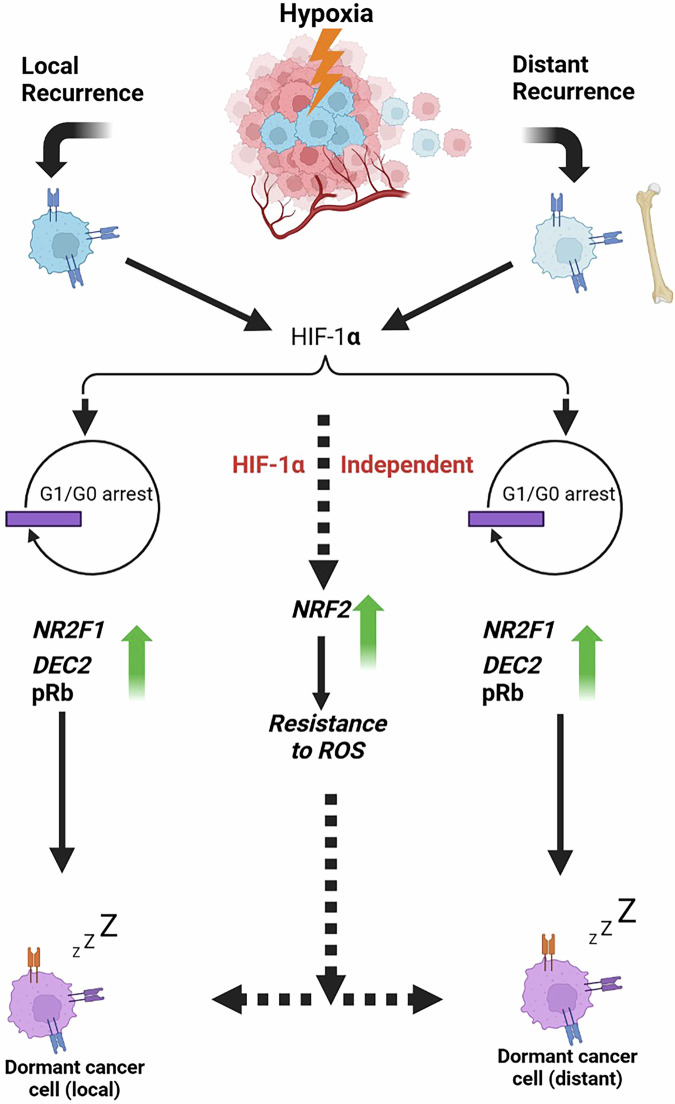
Tumour hypoxia as a major inducer of breast cancer dormancy. Hypoxia is a key driver of breast cancer (BC) dormancy. The presence of hypoxic subregions within the primary tumour is a common feature of BC, arising due to rapid tumour growth exceeding angiogenesis and leading to insufficient oxygen supply. Through both HIF-1α-dependent and independent signalling pathways, hypoxia primes BC cells with stress-adaptive mechanisms that enhance their resilience and survival. This adaptation contributes to the survival of dormant BC cells in the bone marrow, a naturally hypoxic microenvironment. Hypoxia-primed BC cells exhibit a dormant phenotype (G0/G1 arrest) with increased expression of dormancy-associated genes such as NR2F1, DEC2, and post-translational events like pRb, along with activation of the NRF2 oxidative stress response. The well-established link between hypoxia and BC dormancy in disseminated tumour cells (DTCs) may explain the poor prognosis and aggressive recurrence commonly observed in hypoxic BC cases.

Tumour hypoxia is a common and significant feature of BC due to the aberrant vascularisation and poor intratumour blood supply, which reduces oxygen supply to some regions within the tumour [42]. Hypoxic regions within tumours are clinically significant, having an established association with distant metastases, chemo- and radio-resistance, and ultimately a poor prognosis. For example, in a recent study where 1178 women with primary BC were followed up for 15 years for recurrence, patients with hypoxic primary breast tumours were demonstrated to have an increased risk of recurrence [43]. The relationship between hypoxia, metastasis, and recurrence in BC is well-established, such that intravasation of BC cells in the primary tumour is proven to be initiated by hypoxia [44]. Even though there is no definitive answer to how dormancy is induced and established in BC, it is generally agreed that hypoxia plays a role here (Fig. 2).

The hypoxic population of solid tumours has been demonstrated to enter a state of dormancy (G1/G0 arrest) with reduced cellular metabolism as an adaptive survival mechanism [45, 46]. In support of these observations, Fluegen et al. have demonstrated that tumour hypoxia generates a population of dormant DTCs, with a long-lived dormancy and stress-sustaining programme that influences the eventual fate of these dormant DTCs [17]. BC cells in a hypoxic microenvironment have been shown to exhibit high expression of key dormancy genes, including NR2F1, DEC2, and pRb [17, 46]. Hypoxia priming of DTCs in BC may contribute to their enhanced survival in the hypoxic bone marrow microenvironment. The bone marrow is the most favourable homing metastatic site for disseminated dormant BC cells, such that about 60% of ER^+^ BC patients with metastatic relapse exhibit bone metastasis [27, 47, 48]. Although the exact reason for this preferential affinity is not yet known, the hypoxic microenvironment of the bone is believed to support the survival of DTCs and further influences the induction of the proliferative DTCs to enter dormancy [49]. DTCs in patients’ bone marrow have been implicated as the major culprits that eventually re-proliferate into overt bone metastases.

Hypoxia-inducible factor-1α (HIF-1α) signalling has been postulated as key to the establishment of dormant DTC populations in the hypoxic microenvironment [50]. However, it has been demonstrated that BC dormancy in hypoxia can also occur via HIF1α-independent mechanisms [39, 51]. This suggests that different sets of hypoxia-related mechanisms could be responsible for dormancy in hypoxic BC. One such HIF-1α-independent signalling pathway is the activation and signalling of nuclear factor erythroid 2-related factor 2 (NRF2). NRF2 is the master regulator of the cellular antioxidant response [51]. High nuclear NRF2 staining and subsequent increased antioxidant response to oxidative stress has been demonstrated to be critical to the survival and recurrence of BC in preclinical dormancy models [51]. With elevated levels of reactive oxygen species (ROS) associated with hypoxia leading to high oxidative stress, hypoxia-induced dormancy perhaps is an adaptive state of BC cells to survive the associated oxidative stress [52].

Interestingly, the impact of hypoxia on cancer dormancy is not limited to the generation of dormant DTC populations in the hypoxic microenvironment, but also to the fate of these cells as CTCs in blood circulation [53]. Hypoxia priming of DTCs has been shown to increase their resistance to oxidative stress, with improved survival and metastatic efficiency [54–56]. In addition, the downregulation of proliferation markers such as Ki67 and cyclin-dependent kinases (CDKs) [57], and upregulation of the CDK inhibitors p16 and p27 in hypoxic BC cells [58, 59], confirms a key role of hypoxia on dormancy. Hypoxia has been well implicated to trigger intravasation and increase the metastatic ability of BC cells [53]; however, it remains unexplored how hypoxia influences the establishment of long metastatic dormancy in BC.

### Extracellular matrix interactions

**Fig. 3:**
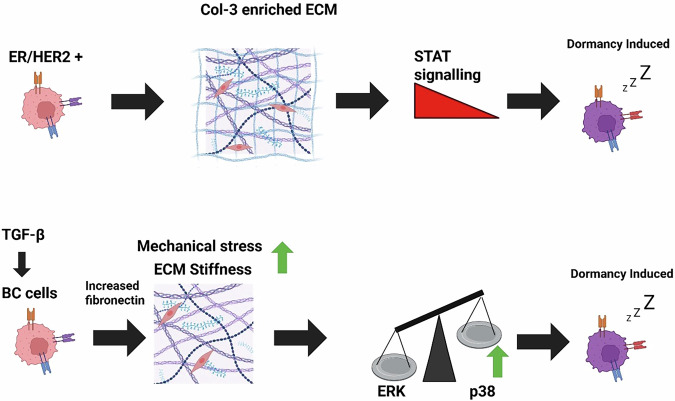
Extracellular matrix (ECM) remodelling and interaction with BC cells induce dormancy. As a major component of the tumour microenvironment, the extracellular matrix (ECM), particularly collagen (Col-3) and fibronectin, plays a pivotal role in inducing dormancy in breast cancer (BC). ECM remodelling in the primary tumour enhances BC cell dormancy through the activation of STAT signalling and ERK/p38 pathways. In addition, collagen-rich ECM, commonly found in tumour hypoxia, increases ECM stiffness. This stiffness restricts cancer cell proliferation by mechanically confining the cells within a limited microenvironment, thereby increasing mechanical stress, which promotes an adaptive dormant state in breast cancer (BC) cells. Thus, the ECM-induced dormancy is considered as an adaptive state of BC cells in response to mechanical stress originating from the tumour microenvironment.

Aside from the hypoxic microenvironment, the interaction of tumour cells with the extracellular matrix (ECM) of the primary tumour is considered as critical to the induction and establishment of dormant BC cells [60]. The ECM is the major non-cellular component of the tumour microenvironment (TME) [61]. Evidence has long been provided that the basement membrane provides a conducive microenvironment that promotes dormancy in BC cells [62]. The impact of tumour ECM on dormancy in BC is component-dependent, such that interaction with different components of the ECM exerts different cellular fates (either to induce dormancy or remain proliferative). For example, several studies have established collagen type III (Col-3) enriched ECM as a major regulator of tumour cell dormancy in BC, particularly in HER2+ and ER+ subtypes [60, 63, 64]. BC cells/Col-3 interaction has been demonstrated to induce and maintain dormancy by disrupting DDR1 kinase-mediated STAT1 signalling [60]. In addition, the remodelling and assembly of fibronectin (ECM protein) via transforming growth factor beta (TGFβ) signalling and ERK/p38 activation has been shown to be key in maintaining dormancy in BC cells (Fig. 3) [65, 66]. Elevated levels of TGFβ are a common feature of the tumour microenvironment and contribute to extracellular matrix (ECM) remodelling by promoting fibronectin production from both stromal and tumour cells [67]. The altered ECM, characterised by increased stiffness and enriched fibronectin content, trigger cellular stress-mediated activation of p38 signalling, a key regulator of dormancy in breast cancer [66, 68]. A delicate balance between the activation (phosphorylation) of ERK1/2 and p38 MAPK plays a critical role in determining whether cancer cells enter a proliferative or dormant state. A high p38-to-ERK activity ratio is a well-established molecular hallmark of dormancy, promoting cell cycle arrest and enhancing the survival of disseminated tumour cells (DTCs) [69–71] Recently, dormant TNBC cells have been demonstrated to be enriched with ECM proteins that induce and sustain dormancy via MAPK signalling pathway [72]. It has been well-documented that the tumour ECM is critical to the survival and treatment-resistant phenotype of BC dormant cells.

The ECM influence on dormancy in BC has perhaps been well described with integrin signalling. However, contrary to the BC cells/Col-3 or fibronectin interactions that induce dormancy, it is the loss of integrin-mediated ECM attachment that induces dormancy in BC cells [73–75]. Integrins are adhesion receptors that are known to transduce signals from the ECM, which are crucial to cell proliferation, survival, and motility [76]; hence, its loss has been shown to promote cell cycle transition from a proliferative to a quiescent state. In BC preclinical models, the loss of integrin beta1, in particular, has been well demonstrated to induce and sustain dormancy by disrupting the integrin β_1_-Focal Adhesion Kinase signalling axis, which is crucial for pro-survival pathways, including phosphatidylinositol 3-kinase (PI3K)/Akt signalling [77, 78]. Although the loss of integrin-dependent anchorage induces anoikis, a form of cell death [79], BC cells employ various other mechanisms, including the induction of an anoikis-resistant phenotype and stromal alterations, to suppress anoikis [80, 81]. These observations are consistent with reported cytostasis associated with integrin inhibitors in BC [82]. The influence of ECM proteins on dormancy induction and sustenance in BC perhaps explains the preferential affinity of dormant BC cells to home in the bone, as osteoblasts and osteoclasts in the bone microenvironment can secrete many ECM-related factors similar to those found in the ECM of breast tumours [83].

Mechanical forces originating from the tumour microenvironment, such as ECM stiffness, are another identified factor that stimulates dormancy induction in BC [84]. Collagen production determines matrix stiffness and regulates the balance between tumour dormancy and proliferation. Tumour hypoxia increases collagen hydroxylation and deposition by both cancer cells and cancer-associated fibroblasts, which stiffens the extracellular matrix [85, 86]. Increased ECM stiffness is considered to restrict the proliferation of cancer cells in a confined microenvironment; hence, dormancy is induced as an adaptive state in response to this mechanical stress [41]. In general, ECM stiffness has been shown to generate dormant subpopulations of cancer cells with enhanced invasive and recurrence ability [87, 88]. Although mechanisms underlying matrix stiffness and BC dormancy remain largely obscure, several ECM-sensing and mechano-transduction pathways have been proposed. For example, in TNBC cells, increased ECM stiffness was demonstrated to induce and sustain dormancy via an epigenetic programme that activates cell-cycle inhibitors, p21 and p27, whilst downregulating integrin b3 [89]. Also, BC cells grown on a stiff matrix induced expression of dormancy genes through the integrin B1-mediated PI3K/Akt pathway in response to the mechanical stress [90]. Breast tumour stiffness is strongly linked to distant metastasis [91, 92], and this may be due to an increase in dormant BC population in response to ECM-induced mechanical stress.

### Therapy-induced stress

In response to treatment, BC cells are now known to induce pro-dormancy programmes as a resistance mechanism to evade treatment. BC dormancy in patients occurring in the therapy setting, either ongoing or completed, has been reported [93]. The induction of dormancy during anti-BC therapy may either be directly through epigenetic and metabolic reprogramming [94–96] or indirectly by creating dormancy-inducing stress factors, such as hypoxia, nutrient deficiencies, and ROS generation, that stimulate the treatment-resistant dormant phenotype [97, 98]. For example, endocrine therapy (ET) has now been established to force BC cells to enter a dormancy state, rather than killing them in HR^+^ BC [94, 99]. A similar observation has been reported in palbociclib (CDK4/6 inhibitor)-treated ER + BC cells [100] and in entinostat (histone deacetylase inhibitor)-treated in breast cancer cells, independent of ER status [95]. In TNBC cells, the use of 5-azacytidine (AZA, a DNA methylation inhibitor) in combination with retinoic acid has been demonstrated to induce dormancy [101]. With the recent discovery of induced cellular dormancy associated with ET and CDK4/6i treatment of BC cells, it is no surprise that most BC patients who receive ET and CDK4/6i eventually relapse, even decades after the treatment period [9, 102]. ET and CDK4/6i is the standard treatment for HR^+^, HER2-ve metastatic breast cancer [102]. The long latency period and subsequent fatal relapse experienced by HR^+^ BC patients is therefore likely due to the induction of a population of dormant BC cells by these targeted therapies [4].

Therapy-induced dormancy in BC is not limited to targeted therapy, as earlier discussed, but also in response to conventional chemotherapy. In TNBC cells, treatments with doxorubicin and cyclophosphamide have been shown to generate a dormant population of TNBC cells, which eventually regrow as metastases post-treatment [103]. Again, neoadjuvant chemotherapy (NAC) in TNBC patients led to a dormant persistent population that was adaptively selected via transcriptional reprogramming [104]. Additionally, in a preclinical mouse model, cyclophosphamide treatment was observed to induce epithelial-to-mesenchymal transition (EMT) of BC cells to generate a persister cell population with a dormant phenotype (reduced proliferation, increased resistance to apoptosis), which was key to metastatic recurrence in the lung post-treatment [105]. Variety of mechanisms underlying therapy-induced dormancy has been reported, although mechanism appears therapy dependent. For example, AZA-treatment of BC cells induce dormancy via a SMAD4-transcriptional programme that restores TGF-β-signalling, which promotes dormancy [101, 106]; however, in metformin-treated BC cells, the induction of dormancy has been attributed to increased mitochondrial respiration and increased oxidative stress [96]. In addition, a global increase in heterochromatin-associated modifications has been reported as responsible for ET-induced dormancy [94, 107]. It is worth noting that these mechanisms are usually not mutually exclusive but co-exist. For example, the activation of the p38-MAPK pathway and the induced expression of p27 often coexist with most other identified mechanisms underlying therapy-induced dormancy [70, 101, 108]. In summary, BC cells induce dormancy as an adaptive protective state to survive different types of chemotherapy or targeted therapy. This significantly contributes to the burden of dormant DTC associated with both early and late recurrence of BC.

## Survival mechanisms of dormant BC

**Fig. 4:**
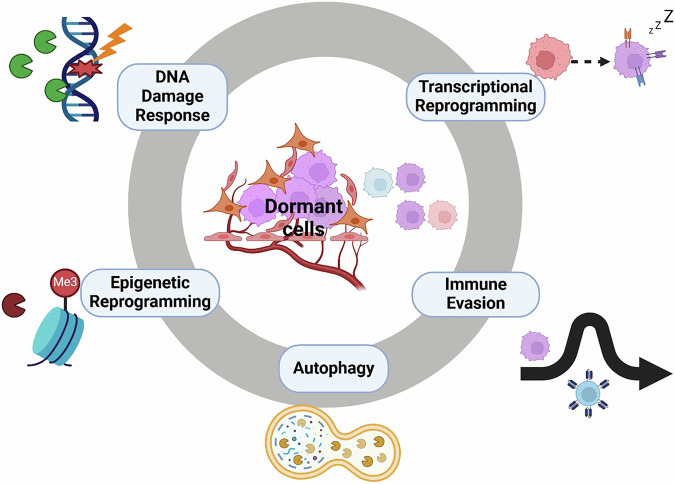
Hallmarks of adaptive survival mechanisms in dormant breast cancer. This summarizes five major adaptive survival mechanisms employed by dormant breast cancer cells. These mechanisms enable the cells to endure extended periods of dormancy and facilitate recurrence when environmental conditions change. Importantly, the mechanisms are not mutually exclusive but are likely to converge on a common intersecting pathway.

Cancer dormancy remains an unmet priority in BC treatment, with an urgent need for novel therapeutics that eradicate dormant cancer cells before the fatal recurrence of the disease. Unfortunately, the adaptive mechanisms that sustain both initial and long-term survival of disseminated dormant BC cells before their recurrence are poorly understood. However, several mechanisms, including stress signalling responses, transcriptional and metabolism reprogramming, post-transcriptional alterations, DNA damage repair (DDR), immunosuppression etc., which are not mutually exclusive, have recently been suggested to be key to the survival and treatment-resistant phenotype of dormant BC cells (Fig. 4). Although these are early days, a better understanding of the biological factors underpinning these dormancy-sustaining mechanisms is critical in developing new therapeutic strategies that eradicate dormant BC populations, thus suppressing their re-emergence as fatal clinical relapse of the disease.

### Transcriptional reprogramming

Transcriptional reprogramming is an early event in cancer dormancy as it is required to induce the genetic programme critical to the induction and maintenance of dormant phenotypes. Cancer dormancy initiates a comprehensive re-writing of transcriptional programmes by selectively activating the expression of pro-dormancy genes whilst suppressing pro-proliferative gene expression [109–111]. This event primes dormant cancer cells with dormancy-surviving programmes, such as autophagy [112], stress-induced p38-regulated transcription factor network [69], EMT programme [104, 113], DNA damage repair [114], and NR2F1-mediated quiescence programmes [109], which are key to the survival, invasiveness, and treatment-resistant phenotype of these cells. For example, dormant BC cells with high affinity for bone metastasis have been shown to exhibit a distinct transcriptional signature via transcriptional reprogramming to activate the expression of prominent BM-associated genes that promote bone metastasis whilst suppressing the expression of a heterogeneous group of genes associated with the primary TME [115]. Additionally, chemo-resistant transcriptional programmes observed in dormant DTC of BC patients after treatment were found not to be pre-existing, but rather acquired through transcriptional reprogramming in response to treatment [104].

The reprogramming of transcriptional activities in cancer dormancy is critical to the survival of dormant cells, such that the genetic signature of the dormancy in BC is considered strongly predictive of a metastatic phenotype [116]. Interestingly, the resultant genetic profile of individual dormant cancer cells after transcriptional reprogramming is diverse and heterogeneous depending on the tumour type and a patient’s genetic makeup. For example, the molecular characterisation of circulating dormant cells from the same blood sample of a BC patient demonstrated heterogeneous subpopulations with different transcriptional profiles [117, 118]. The heterogeneous nature of the resultant transcriptional profiles of dormant breast cancer (BC) cells post-reprogramming reflects the clinical heterogeneity among metastatic lesions, which remains a challenge to treatment [119].

#### Global transcriptional repression

With this said, the reprogramming of transcriptional activities, despite the diverse resultant dormancy-sustaining signatures and programmes of individual dormant BC cells, converges on global repression of the transcription machinery [111]. The reduction in the rate of global transcription (global transcriptional repression) is a common and key feature of all transcriptional reprogramming in cancer dormancy [109, 111], which enables dormant cells to remain transcriptionally active yet at a reduced rate compared to proliferating cell [120]. The occurrence of transcription repression is evident in dormant cancer cells with the induction of global chromatin repression and condensation [109, 120], although its significance as a survival mechanism for dormant cancer cells remains elusive.

However, in dormant yeast models, global transcriptional repression has been demonstrated as an essential mechanism required for the longevity and reversibility of the dormant yeast, as it allows only the expression of genes that promote survival whilst maintaining low transcription-associated DNA damage [120, 121]. The inhibition of global transcriptional repression in these cells induced permanent arrest with a shortened lifespan [121]. With the repression of transcriptional activity in dormant yeast demonstrated to be similar to their mammalian counterparts [122], global transcription repression could potentially be an adaptive survival mechanism of dormant BC cells, although the mechanism underlying this process during cancer dormancy remains unknown.

### Autophagy

Autophagy is a well-established adaptive survival mechanism of cells by which cellular components are recycled to generate energy in response to cellular stress, such as DNA damage, hypoxia, nutrient deprivation, and other metabolic stresses [123]. As a stress signalling response, the activation of autophagy is central to tumour development. It has been described as being key to the progression and treatment-resistant phenotype of many tumours, including BC [124]. The induction of autophagy in BC dormancy is well demonstrated and has been long proposed as a potential survival mechanism of dormant cancer cells [112]. Although little experimental evidence initially supported this, a growing body of evidence in recent times now seems to establish autophagy as critical to the survival of disseminated dormant breast cancer cells [46, 125–127]. The occurrence of autophagy has now been demonstrated and validated in various experimental BC dormancy models, including hypoxia-induced [46], ECM-induced [127] or therapy-induced [125, 128] dormancy models. In these models, the induction of autophagy in dormant breast cancer (BC) cells has been shown to occur through various mechanisms. These autophagy-inducing mechanisms include the activation of PTEN-induced putative kinase protein 1 (PINK1) in response to increased oxidative stress induced by high ROS levels in dormant BC cells [52, 127], activation of AMP-activated protein kinase (AMPK) in response to ECM detachment and hypoxia [129, 130], and reduced PI3K–AKT signalling in response to nutritional stress [131]

Autophagy has been demonstrated in many preclinical models as a critical survival mechanism for dormant breast cancer (BC) cells, with the pharmacological and genetic inhibition of autophagy leading to the death of dormant BC cells and the subsequent inhibition of tumour recurrence. Additionally, autophagy has been shown to protect dormant BC cells by inducing chemo-resistant phenotypes leading to the survival of dormant BC cells during treatment [132]. On the contrary, in dormant BC stem cells, the inhibition of autophagy was observed to drive the escape from metastatic dormancy as well as activate the proliferative programmes of BC stem cells (tumour relapse) [112], suggesting autophagy as an anti-proliferative mechanism of dormancy rather than an adaptive survival mechanism as earlier stated. To explain this contradiction, Aqui et al. demonstrated that it is the genetic inhibition of autophagy via ATG5 deletion that elicits dormancy escape but not pharmacological inhibition of autophagy using hydroxychloroquine [125]. With the role of autophagy in dormancy still largely elusive, it would be essential to identify autophagy mechanisms that either support survival or maintenance of quiescent phenotypes of dormant BC cells, as this will be critical in the consideration of which pathway can be targeted as a therapeutic strategy. Interestingly, the role of autophagy in dormancy is not limited to cancer dormancy as it has also been shown to support the survival of nematode larvae in their dormant state [133]. This further underscores the significance of autophagy in the survival of quiescent cells such as BC dormant cells.

#### Clinical significance of autophagy in BC dormancy

With these encouraging pre-clinical findings, the inhibition of autophagy as a therapeutic strategy to eliminate dormant breast cancer (BC) cells is currently being assessed in several clinical trials (NCT03032406; NCT04841148; NCT0452385). These trials are evaluating the efficacy of the autophagy inhibitor hydroxychloroquine either as a monotherapy or in combination with a checkpoint inhibitor (avelumab), a CDK4/6 inhibitor (palbociclib/ abemaciclib), or an mTOR inhibitor (everolimus) to eliminate dormant cancer cells in BC patients who are in remission but harbour DTCs in their bone marrow. Although publication of this data is currently eagerly awaited, a preliminary report from one of these ongoing trials in TNBC patients (NCT03032406) suggests that hydroxychloroquine and everolimus effectively reduced DTC burden (80% reduction) with only 2/53 patients experiencing disease recurrence after a median follow-up of 42 months (range 7-60 months) [134]. In summary, the current data, although limited, appear to position autophagy as a critical survival mechanism for dormant breast cancer (BC) cells and suggest that inhibiting autophagy may be a potential therapeutic strategy to suppress BC recurrence by eradicating residual dormant cancer cells.

### DNA damage repair mechanisms

Breast cancer cells with a dormant phenotype exhibit highly efficient DNA damage repair (DDR) machinery, with upregulation of several DDR pathways which contribute to their chemo- and radio-resistant phenotype [135–138]. The activation of DDR mechanisms is crucial for the survival of dormant cancer cells following primary treatment. The DDR machinery is a highly complex network of mechanisms that detect and repair DNA damage to maintain genomic stability and integrity. Hence, the effectiveness of chemo-and radio therapies as cancer treatment is dependent on the inability of cancer cells to efficiently repair the high levels of DNA damage inflicted on them by these therapies [139]. However, enhanced DDR activity is reported in many dormant cancer cells, including BC [136], such that DDR signalling, particularly the ATR-Chk1 pathway (a key DDR mechanism) in a cohort of invasive BC patients, was shown to be predictive of early local and distant recurrence [140]. This is suggestive of an essential role of DDR in the survival of dormant BC cells, considering that dormant BC cells are responsible for BC recurrence. In addition, expression of DDR proteins, ERCC1 [141] and RAD51 [142], in dormant CTCs in blood samples of metastatic BC patients has a significant association with therapy failure, which is suggestive of enhanced DDR capacity, particularly base excision and homologous recombination repairs. Non-homologous end joining repair has also been implicated in facilitating DNA damage repair in ER+ dormant breast cancer cells in response to chemotherapy and radiotherapy treatment [138]. Consistently, the inhibition of the DDR pathway has been shown to sensitise dormant BC cells to chemotherapy such that the combination of DDR inhibitors to current chemotherapy has been proposed as a potential strategy to eliminate dormant BC cells [137]. In support of this, an inhibitor of PARP (a DDR protein) was observed to significantly reduce recurrence risk by 42% and prevent progression to metastatic disease among high-risk, HER2-negative BC patients [143]. Just as with most active proteins in BC dormancy, the exact role of PARP in the survival of dormant BC cells remains unknown, although its inhibition appears to hold therapeutic potential in eliminating dormant BC cells, which in turn reduces metastatic recurrence.

### DDR and oxidative stress in BC dormancy

Resistance to oxidative stress is common features of dormant cancer cells, which some have suggested may be due to the high expression and activity of antioxidant elements such GPX4, ALDH, and NRF2 in these cells [54, 144]. However, the increased activation of DDR mechanisms in BC dormant cells even in the absence of DNA damage may perhaps contribute to the resistant phenotype of dormant BC cells to oxidative stress. Dormant DTCs are highly sensitive to intracellular ROS activation, a series of molecular mechanisms, aiming to control excessive oxidative stress [145]. Loss of DDR players such as NuMA and ATM has been shown to increase oxidative DNA damage in quiescent cells like neuron [146]. However, this phenomenon is yet to be studied in dormant cancer cells. Interestingly, in normal quiescent cells, PARP1 has recently been reported to play a key role in the repair of oxidative DNA breaks and cell survival by binding to chromatin through NuMA following oxidative DNA damage to cells [147]. With high levels of ROS in dormant BC cells, PARP1 could play a critical role in the continuous repair of oxidative DNA damage, potentially contributing to the resistance of dormant BC cells to oxidative stress. However, this has yet to be tested. Coincidentally, PARP inhibition has been shown to significantly reduce the incidence of recurrence in BC patients. These observations reinforce the potential yet unexplored role of DDR players and oxidative stress in dormant BC cells [143].

The expression of DDR proteins in dormant BC cells has been suggested to occur in response to DNA damaging treatment [114]. However, an increased expression of DDR proteins has been reported in yeast dormancy models in the absence of DNA damage [121]. In this model, the expression of DDR protein Rad93 (human homologue CHK2) was shown as a key checkpoint in the induction and maintenance of dormancy/quiescence in response to replicative stress (not DNA damage) such that the inhibition of this checkpoint led to apoptotic cell death. Additionally, Rad93 was demonstrated to be key to the transcriptional reprogramming of these cells during dormancy [121]. It remains to be elucidated if these mechanisms are relevant to the survival of dormant BC cells.

### Epigenetics mechanisms

Dormancy-induced epigenetic programmes, particularly DNA methylation and histone modifications, have been well implicated in both the survival and subsequent re-proliferation of dormant BC cells. Epigenetic alterations are common in BC, particularly HR^+^ subtypes, and are suggested to contribute to the long-term dormancy associated with this disease [148]. For example, ET-induced dormancy in HR^+^ BC patients is characterised by a consistent epigenetic reprogramming which includes a global increase in histone repressive marks (H3K9me2, H3K27me3, and H4K20me3) [107]. Interestingly, H3K27me3-associated polycomb repressors are key in the global transcriptional repression of genes, particularly notch-related genes in dormancy [149]. Consequently, the addition of epigenetic therapy to the current treatment of BC is being investigated in BC patients (NCT02115282, NCT01935947, NCT01928576, NCT02453620) as a new strategy to eliminate dormant BC cells and prevent a recurrence.

Tumour hypoxia, ECM stiffness and antiproliferative therapies have been reported to trigger and sustain dormancy in BC through diverse epigenetic programmes which mediate the activation of different dormancy-sustain programmes, leading to heterogeneous populations of dormant BC cells [89, 103, 150] The epigenetic landscape of dormant BC cells is complex, and little is currently known about the definite epigenetic mechanism responsible or critical to the survival of dormant cancer cells. However, increased hypermethylation of genes including the tumour suppressor genes *CST6, SOX17, and BRMS1* has been shown in dormant CTCs from BC patients, particularly with metastatic disease [37]. In addition, the upregulation and activation of NR2F1-mediated dormancy programmes, which included the transcriptional activation of histone repressive marks (H3K4me3 and H3K27ac) and the deacetylation of histone H3, is epigenetically upregulated [109]. The upregulation of Tet-2, epigenetic enzyme, and subsequent generation of 5-hydroxymethylcytosine has been shown to be associated with the maintenance of dormancy in BC cells [89].

Despite these insights, it remains to be determined whether the epigenetic reprogramming in dormant cancer cells promotes anti-proliferative mechanisms of dormancy rather than an adaptive survival mechanism, as the direct inhibition of dormancy-induced epigenetic programmes does not eliminate the population of dormant BC cells but rather prolongs their latency period of dormancy. For example, the inhibition of histone deacetylase in BC cells was shown to induce a promotion of dormancy (not cell death) through epigenetic reprogramming [95]. However, inhibiting histone methyltransferase, EHMT2, was observed to prevent the development of dormancy in BC cells and further eradicate dormant BC cells by reversing their dormancy-induced epigenetic programme [107]. It is possible that the epigenetic reprogramming of dormant BC cells complements their transcriptional reprogramming to activate various dormancy-surviving programmes, including autophagy, immunosuppression and DDR mechanisms. For example, this inhibition of autophagy in dormant BC cells using hydroxychloroquine also deregulates DNA methylation and DDR mechanisms of these cells [137, 151]. These reports suggest that epigenetic reprogramming in dormant breast cancer (BC) cells may play a critical role in both the induction and maintenance of dormancy, as well as in subsequent adaptive survival mechanisms.

### Immunoevasion

The immunosurveillance of cancer cells by the hosts’ immunity is a barrier to the successful dissemination and subsequent metastatic colonisation of cancer cells. However, dormant cancer cells, particularly those in circulation, are now known to evade this immunity through various immunoevasive mechanisms, thereby advancing their survival and progression [152]. Immunoevasion is considered as an emerging hallmark of cancers, although its generality remains to be established among human cancers [153]. Nevertheless, its significance has been established as key to the progression of BCs. For example, the suppression of the innate immunity (interferon pathways in particular) by BC cells has been shown to enhance escape from immunosurveillance, thus increasing CTC population in circulation and promoting bone metastasis [154]. In addition, BC patients with a suppressed expression of genes associated with innate immune responses developed a higher number of bone metastases [154].

Dormant BC cells are now known to be primed with several immune-escape properties in the primary TME prior to their dissemination. In addition, the expression of programmed cell death ligand 1 (PD-L1) is upregulated in hypoxic cancer cells as an immune escape from cytotoxic T-cells [155]. Similar observations have been reported with EMT- and drug-induced dormancy [156, 157].

Consequently, dormant BC cells, whether within the primary tumour or in circulation as CTCs or at the site of colonisation, exhibit diverse immunosuppressive and immunoediting properties which promote their survival. Dormant populations of TNBC cells have been shown to resist T cell attack by forming an immunosuppressive cluster through activation of a hypoxia-induced programme [158]. Dormant DTCs of BC in the lungs are also reported to avoid immune attack by recruiting and accumulating neutrophils to create immunosuppressive niches [159]. CTCs from BC patients, regardless of HR or HER2 status, frequently express PD‑L1 expression as a means to avoid their elimination by T cells in circulation [160]. This high PD-L1 expression on CTCs from BC patients has been suggested as a useful biomarker for immune checkpoint therapies like PD-1/PD-L1 inhibitors. However, resistance to PD-L1 checkpoint therapy has been reported in BC patients despite the high expression of PD-L1 on CTCs [161], suggesting that CTCs may possess compensatory immune-escape mechanisms beyond PD-L1 expression that promotes their survival. In HER2^+^ BC, dormant DTCs, through inhibiting the WNT signalling pathway, supress ligands for natural killers to evade innate immunity [162]. Aside these immune-escape mechanisms, circulating dormant BC cells are also known to mechanistically aggregate with platelets to avoid immune detection in the circulation [163]. With all this said, the immunoevasion mechanisms of dormant BC cells remain complex, with further studies required to understand this adaptive survival mechanism fully. Insight into these mechanisms hold significant potential to target BC dormancy before the occurrence of overt distant metastases.

## Impact of the metastatic niche on breast cancer dormancy

In addition to the discussed adaptive mechanisms that enable the long-term maintenance and survival of dormant disseminated breast cancer (BC) cells, their eventual capacity to persist, colonize, and recur in distant organs such as the lung, liver, and bone (Fig. 1), is ultimately governed by the organ-specific characteristics of the metastatic niche. These niches provide specialised microenvironments that can be either hospitable or hostile to the survival of dormant BC cells that arrive in these distant organs. For example, whilst the bone microenvironment provides endothelium, osteoblasts, and hematopoietic stem cells, which support the colonisation and survival of dormant BC cells [164], the high levels of oxidative stress in skeletal muscle suppress their colonisation and survival [165]. Thus, the bone represents a favourable site for BC metastasis, whereas skeletal muscle remains largely inhospitable to these cells. Even more importantly, the microenvironment of the metastatic niche can actively induce dormancy in proliferative BC cells upon their arrival. In the lungs, interactions between disseminated proliferative BC cells and resident alveolar macrophages have been shown to induce dormancy through TGF-β2 signalling [166]. Also, the high levels of retinoic acid in addition to hypoxic nature of the bone marrow has been demonstrated to induce dormancy in proliferative BC cells [39, 109, 167]. Thus, the dormancy-inducing and -sustaining capacity of these metastatic microenvironments plays a critical role in regulating the dormancy and long-term survival of disseminated BC cells.

With this said, although the induction and maintenance of BC dormancy at various metastatic sites largely results from interactions of BC cells with the foreign microenvironment at the distant organ, the adaptability of these cells likely reflects their plastic phenotype that is either acquired or preconditioned by the primary tumour microenvironment, as earlier discussed [168]. This acquired plasticity enables dynamic phenotypic transitions and functional flexibility, allowing disseminated breast cancer (BC) cells to respond to diverse microenvironmental factors within various metastatic niches and to persist in a dormant state [169, 170]. It’s worth noting that, although the mechanisms underlying dormancy induction in the primary tumour may differ from those in secondary metastatic sites, dormant BC cells often share similar adaptive survival mechanisms: autophagy, transcriptional and epigenetic reprogramming, immune evasion, and DDR (Fig. 4) to resist apoptosis and maintain the dormant phenotype across both environments [111, 171].

Perhaps the most crucial role of the metastatic niche in breast cancer biology is its key function in reactivating dormant disseminated tumour cells (DTCs), a significant challenge for improving patient outcomes. While the microenvironment of various metastatic niches does support the long-term, clinically undetectable dormancy of disseminated BC cells, specific changes and signals are known to trigger dormancy escape and promote rapid metastatic outgrowth. In truth, it remains incompletely understood which definitive factors and mechanisms drive the reawakening of dormant breast cancer cells across metastatic sites. However, inflammatory cytokines and neutrophils [172–174], activated macrophages [166, 175], and age-related changes in the microenvironment of these metastatic sites [176–178] have been strongly implicated. For example, reduced oestrogen, a hallmark of menopause, has been shown to increase the expression of angiopoietin-2 and interleukin-6 (IL-6) in the bone, both of which can activate the growth of dormant DTCs [179, 180]. Similarly, age-induced increases in platelet-derived growth factor (PDGF) expression in the ageing lungs have been shown to stimulate dormant BC cell reawakening and drive relapse of ER+ breast cancer in the lung [176]. Inflammatory cytokines released by various metastatic niches, whether triggered by injury, smoking or disease, are increasingly recognised as major contributors to BC recurrence. However, the mechanistic understanding of this process remains in its early stages [178, 181, 182].

Collectively, changes in the microenvironment of BC metastatic niches have the potential to override intrinsic or therapy-induced dormancy signals in dormant BC cells, reactivating their proliferative programmes and driving the aggressive outgrowth of clinically detectable metastases. Therefore, preclinical models that deepen our understanding of strategies to eliminate dormant breast cancer cells, rather than merely maintaining them in a dormant state either in the primary tumour or metastatic niches, are essential for preventing relapse and improving long-term patient outcomes.

## Preclinical models in BC dormancy

Experimental models remain essential for understanding breast cancer (BC) dormancy and identifying clinically relevant targets. Several in vitro and in vivo models have been used to investigate various dormancy survival mechanisms in different dormancy-inducing settings. While a comprehensive overview of these models has recently been reviewed [183], Table 1 highlights a few representative examples. It is important to note that some dormancy mechanisms identified in experimental models, particularly in vitro models, are yet to be observed in clinical settings [111], underscoring the need to design new models that better reflect the biology of clinical dormancy. Unfortunately, this is not the case, as most in vitro dormancy models primarily focus on either therapy-induced or ECM remodelling-induced dormancy in BC cancer.

**Table 1:** Examples of reported BC dormancy models used to study various dormancy survival mechanisms.

Mode of dormancy induction	Model type	Described survival mechanism
Therapy induced (5-azacytidine and retinoic acid)	in vitro (2D)	Transcriptional reprogramming [101]
Therapy induced (aromatase inhibitor-mimicking)	in vivo (mouse)	Metabolic and Transcriptional reprogramming, Autophagy [96]
Therapy induced (tamoxifen)	in vitro (2D)	Transcriptional [190] and Epigenetic [107] reprogramming
Hypoxia	in vitro (2D)	Metabolic reprogramming, Autophagy [46, 184]
Hypoxia	in vivo (mouse)	Transcriptional reprogramming [17]
Extracellular matrix interactions	in vitro (3D)	Transcriptional reprogramming [191]
Extracellular matrix interactions (metastatic niche)	in vitro (2D) & in vivo (mouse & zebrafish)	Transcriptional reprogramming [192]
Extracellular matrix interaction	in vitro (2D) & in vivo (mouse & chicken eggs)	Transcriptional reprogramming [60]
Extracellular matrix interaction	in vitro (3D) & In vivo (mouse)	Transcriptional reprogramming [193]
Metastatic niche (bone marrow)	in vitro (co-culture, 2D) & in vivo (mouse)	Autophagy [194]
Metastatic niche (lungs)	in vivo (mouse)	Immune evasion and Cytokine signalling [195]

Specific cytokines and growth factors, as well as serum deprivation, have often been employed to induce dormancy in these models [183]. However, the inclusion of hypoxia is often lacking in most reported models. Out of 94 in vitro BC dormancy studies published in the past 20 years, only two have incorporated hypoxia [46, 183, 184]. Surprisingly, hypoxia in the primary breast tumour has now been shown to programme BC cells with a dormancy phenotype that persists in metastatic cells at various non-hypoxic sites of recurrence [17], emphasising the importance of hypoxia on breast cancer dormancy. This hypoxic priming is particularly relevant to the bone marrow, a common site of breast cancer metastasis, which is inherently hypoxic [27]. The bone marrow microenvironment appears to provide a supportive niche that favours the survival and maintenance of these preconditioned dormant cancer cells. Even more importantly, disseminated dormant BC cells preconditioned by hypoxia in the primary tumour have been shown to survive better in non-hypoxic metastatic niches, such as the lungs, compared to non-hypoxia-exposed dormant BC cells [17]. This observation highlights a critical gap in current models that neglect the influence of hypoxia. Admittedly, BC cancer dormancy is a complex phenomenon, and the lack of experimental models, particularly in an in vitro setting, that accurately recapitulate the in vivo setting and clinical realities of the disease remains a significant challenge. However, the inclusion of factors critical to the clinical biology of disseminated BC cells, particularly hypoxia, is more likely to model clinical dormancy and offer a more clinically relevant understanding of BC dormancy.

## Conclusion and perspectives

Breast cancer recurrence remains a major clinical challenge in BC treatment. Although several theories have been proposed to explain this phenomenon, the molecular mechanisms underlying breast cancer (BC) recurrence are currently poorly understood, thus limiting the development of novel therapeutic solutions. Growing evidence in recent years, as we have discussed, strongly implicates dormant BC cells as the culprit for both early and late BC recurrence. Hence, targeting dormant cancer cells is considered a promising strategy in preventing cancer recurrence. Unfortunately, no dormancy-specific therapy has yet been approved. However, three potential therapeutic strategies have been suggested and are currently being attempted [185], namely:

- Strategies to maintain dormant cancer cells in a perpetual dormant state – This strategy is considered the most popular as it appears to explain the clinical successes of anti-oestrogen therapeutics and CDK4/6 inhibitors (dormancy-inducing agents), which support long-period remission and prevent recurrence [186]. Unfortunately, the sudden re-proliferation of dormant BC cells into metastatic lesions, as is now known, is not necessarily driven by the activation of something new within the dormant cells, but rather as a response to microenvironmental changes within the homing organ [176, 187]. This discovery thus discredits this strategy and unravels the awaiting danger of sudden BC recurrence in response to microenvironmental changes despite the presence of these dormancy-inducing agents.
- Strategies to intentionally reactivate dormant cells and target their proliferation with current anti-proliferative agents—This strategy has been suggested and demonstrated in other cancers (not BCs) to improve the response to chemotherapy [188, 189]. However, this is considered a very risky approach as failure of the reactivated dormant cells to respond to anti-proliferative therapy could worsen the outcome of the disease
- Strategies to eradicate dormant cancer cells are considered as the most promising yet challenging approach with the inherently acquired treatment-resistant phenotype of dormant BC cells. However, growing preclinical and clinical evidence suggests a strong correlation between the inhibition of the adaptive survival mechanisms of dormant cells and the depletion of these cells. The challenge of this strategy remains with the certainty of complete eradication of dormant cells as surviving dormant cells, post-dormancy-specific treatment, could transform into aggressive disease. A better understanding of the adaptive survival mechanisms, as comprehensively discussed, could assist with the development of novel therapeutics capable of completely eradicating DTC cells both before and after primary treatment.

The development of novel therapeutics aimed at depleting the DTCs and CTCs population in BC patients, in our view, is the preferred approach to preventing recurrence and improving BC treatment. However, the specificity of the discussed survival mechanisms to cancer dormancy remains to be answered as dormancy-sustaining mechanisms also have significant functions in normal homoeostasis. For example, although the inhibition of autophagy [134] and DDR mechanisms [143] appear to successfully demonstrate the clinical significance of inhibiting dormancy-sustaining mechanisms in the eradication of minimal residual disease (MRD), it remains to be determined whether extended administrations of these therapies, which perhaps is required for complete MRD eradication, will be accompanied by additional toxicities. Additionally, the generality and compensatory nature of these mechanisms across BC subtypes remain unknown, and it would be prudent to establish this to stratify patients likely to benefit from MRD eradication agents. Ultimately, the identification of a dormancy-specific pathway, which is critical to the survival of dormant DTC regardless of their BC subtype and its dormancy-inducing conditions, would be valuable in treating the current incurable phenomenon of BC recurrence.
